# Phylogenomics of prokaryotic ribosomal proteins

**DOI:** 10.1186/gb-2011-12-s1-p30

**Published:** 2011-09-19

**Authors:** Natalya Yutin, Kira S Makarova, Yuri I Wolf, Eugene V Koonin

**Affiliations:** 1 National Center for Biotechnology Information, National Library of Medicine, National Institutes of Health, Bethesda, MD 20894, USA

## Background

Archaeal and bacterial ribosomes contain more than 50 proteins. Thirty-four ribosomal proteins (r-proteins) are universally conserved in the three domains of cellular life (Bacteria, Archaea and Eukarya), and 33 r-proteins are shared between Archaea and Eukarya to the exclusion of Bacteria; there are also 23 Bacteria-specific, 1 Archaea-specific and 11 Eukarya-specific r-proteins [1]. Despite the high sequence conservation of r-proteins, the annotation of r-protein genes is often dificult because of their short lengths and biased sequence composition.

## Methods

To perform a comprehensive survey of prokaryotic r-proteins, we developed an automated computational pipeline for the identification of r-protein genes and applied it to 995 completely sequenced bacterial genomes and 87 archaeal genomes available in the RefSeq database. The pipeline employs curated seed alignments of r-proteins to run position-specific scoring matrix (PSSM)-based BLAST searches against six-frame genome translations, thus overcoming possible gene annotation errors. Likely false positives are identified using comparisons against the original seed alignments.

## Results

In the course of this analysis, we gained insight into the diversity of prokaryotic r-protein complements, such as missing and paralogous r-proteins and distributions of r-protein genes among chromosomal partitions. A phylogenetic tree was constructed from a concatenated alignment of 50 almost-ubiquitous bacterial r-proteins. The topology of the tree is generally compatible with the current high-level bacterial taxonomy, although we detected several inconsistencies, possibly indicating uncertain or erroneous classification of the respective bacteria. Similarly, a concatenated alignment of 57 ubiquitous archaeal proteins was used for an archaeal phylogenetic tree reconstruction. In both Bacteria and Archaea, the patterns of the presence/absence of non-ubiquitous r-proteins suggest several independent losses and/or gains of these proteins. According to parsimony reconstruction, three bacterial and five archaeal r-proteins do not appear to be ancestral. Remarkably, all five non-ancestral archaeal r-proteins are present in Eukarya.

## Conclusions

Extended sets of prokaryotic r-proteins were created. Alignments of these sets may be used as new seed profiles for the identification of r-proteins in new genomes and for comparative genomics studies.

## References

[B1] Lecompte O Ripp R Thierry JC Moras D Poch O Comparative analysis of ribosomal proteins in complete genomes: an example of reductive evolution at the domain scale Nucleic Acids Res 2002 30 5382 5390 10.1093/nar/gkf693 12490706 PMC140077

